# Endocrine Disruptors in a New Era of Predictive Toxicology and Dealing With the *“More is Different”* Challenge

**DOI:** 10.3389/ftox.2022.900479

**Published:** 2022-04-27

**Authors:** Terje Svingen

**Affiliations:** National Food Institute, Technical University of Denmark, Kongens Lyngby, Denmark

**Keywords:** emergent properties, adverse outcome pathway (AOP), reproductive toxicology, chemical risk assessment, new approach methodologies (NAMs), endocrine disrupting chemicals

## Abstract

Environmental chemicals, including endocrine disrupting chemicals (EDCs), pose a threat to human health. Actions are taken by scientists, assessors, regulators, and policymakers around the world to improve testing strategies for chemical substances, including pushing towards greater reliance on data from new approach methodologies to replace animal toxicity studies. This paradigm shift is envisioned to ultimately replace animal testing altogether for many purposes. As regards identification and regulation of EDCs, this poses certain challenges in that current guidelines—at least within the European regulatory framework—stipulate that adverse outcomes are to be demonstrated in an intact organism. The new testing paradigm is, of course, to find ways of dealing with this dilemma. However, another challenge still remains, even if the “intact organisms” definition changes or is replaced, namely the challenge of predicting apical adverse effects resulting from endocrine disruption. The adverse outcome pathway (AOP) framework provides a good platform for identifying and regulating EDCs based on both non-animal and animal (or human) data, but also here we are confronted with the same challenge: how to predict adverse effects in complex organism from simple test assays that are based on reductionist principles? In this article, the challenge of “emergent properties” in predictive toxicology is highlighted as a cautionary footnote because, although a future relying far less on animal toxicity testing is both desirable and sensible, the pace at which we transition to the new paradigm should ensure that human health, and the environment, is safeguarded from harmful chemical substances.

## Introduction

Huge efforts are currently being put towards developing non-animal testing strategies for assessing and regulating chemicals, not least within the European Union (EU, 2010; Knight et al., 2021; Pistollato et al., 2021; Carusi et al., 2022). The end goal is not necessarily to completely eliminate animal toxicity testing, at least not in the short term, but rather to significantly reduce the numbers that are used. This strategy is judicious, in that it will enable the testing and assessing of a much greater number of chemical substances in a shorter time at a lesser cost. This strategy is also principled, in that it can greatly reduce the number of animals that need to be expended in order to safeguard human health and wildlife from environmental pollutants. This strategy is not without challenges, however, which should be kept in mind when we charge towards a new future of toxicological testing of industrial chemicals.

One of the big challenges with the predictive toxicology paradigm is, in my opinion, accounting for “emergent properties” of complex systems. To explain this in more detail, I will in the following sections lean on the adverse outcome pathway (AOP) concept, particularly since this framework also offers great opportunities to improve on the predictive power of alternative test methods and strategies. I will also narrow the discussion to endocrine disrupting chemicals (EDCs), for two reasons. Firstly, because the AOP framework is very well suited for addressing the current regulatory needs for identifying EDCs. Secondly, because EDC identification represents an area of chemical toxicology where predicting *in vivo* effect outcomes using data from new approach methodologies (NAMs) is challenging, as we recently argued (Svingen et al., 2022).

To return to the concept of “emergent properties”, I begin by citing a 50 year old article by the physicist and Nobel laureate P.W. Anderson published in *Science* back in 1972. In this now classic article with the three-word title *More Is Different*, Anderson argues that even though we can understand complex systems by breaking it down into smaller units (or laws), we cannot just as easily use our knowledge of these smaller units to construct complex systems. Or to quote: *“The ability to reduce everything to simple fundamental laws does not imply the ability to start from those laws and reconstruct the Universe”* (Anderson, 1972). Regarding modern toxicology and, for instance, the AOP framework or EDC identification, this cautioning from the past is worth noticing.

The following sections will briefly outline how current EDC testing strategies aligns well with the AOP concept and focusing on the opportunities at hand; both with respect to reducing animal testing in chemical toxicity testing more broadly and the more immediate opportunities for scientists and regulators that lies in developing AOPs. Hopefully, this will highlight the fact that we can, and likely will, achieve much in a short space of time. But first, how to define an EDC.

## Endocrine Disrupting Chemicals

As the name implies, an EDC is a chemical substance that can disrupt normal hormone action. However, as recently discussed (Vandenberg, 2021), there is no universal definition for an EDC and various definitions are used by different agencies across the world. The most widely accepted definition, and the one adhered to herein, is the one stipulated by the World Health organization (WHO)/International Programme on Chemical Safety (IPCS), which states that an EDC is “*an exogenous substance or mixture that alters function(s) of the endocrine system and consequently causes adverse health effects in an intact organism, or its progeny, or (sub) populations*”.

A key point that needs further elaboration is the inclusion of “*adverse health effects*” in the definition. By this, it is understood that it is not enough to show that a chemical substance has the potential to disrupt hormone action by, for instance, *in vitro* data or altered hormone profiles in intact animals. Rather, adverse effect must also be shown in an intact organism (e.g., animal). Incidentally, this inclusion introduces another challenge in that “adversity” itself does not have a universal, agreed-upon, definition.

The IPSC defines an adverse health effect as “*a change in morphology, physiology, growth, development or lifespan of an organism which results in impairment of functional capacity or impairment of capacity to compensate for additional stress or increase in susceptibility to the harmful effects of other environmental influences*”, as discussed in (Vandenberg, 2021). This definition appears simple enough, yet what constitutes an ‘*intact organism*’ in the context of toxicity testing is not clear and still debated. A 2017 consensus statement from a panel of experts argued that intact organisms could also include, for instance, surgically and genetically modified animals (Solecki et al., 2017). This opens up for more possibilities than what would follow from a strict interpretation of the dictionary entry of the term, which instead would preclude any organism that have been changed from their natural state. This issue clearly deserves clarification. Nevertheless, a prevailing challenge with current EDC definitions is that it is exceedingly difficult to prove that any substance is an EDC to a level it becomes regulated. For an EDC classification, risk assessors need to provide substantiated evidence for three levels where a proven adverse outcome in an intact animal ranks the highest. In addition, both an endocrine mode of action and a biologically plausible link between the mode of action and the adverse outcome is to be substantiated, at least according to current EU criteria.

Because of these stringent criteria, very few substances are in fact (to date) regulated based on their EDC properties (Svingen et al., 2022), which leads on to a third challenge. With the emerging paradigm of relying more on NAMs for chemical risk assessment purposes, there is an obvious challenge with the “adverse effect in an intact organism” criterion. How will we go about “proving” this if we are not to use animals for toxicity testing? This is still not clear, but will most likely, in an interim period at least, mean that alternative methods are used to a greater extent to screen and prioritize chemicals substances for full toxicity testing, including *in vivo* testing. But this challenge may also mean that we need to redefine what constitute an EDC in a regulatory sense. Sharpening the EDC definition or not, at the very least we need to provide much more information about mechanisms of causality from initial chemical perturbation to adverse health effects if we are to rely more heavily on alternative test method data for predicting *in vivo* effect outcomes. This can be done in many ways, one being the AOP framework, as discussed next.

## Adverse Outcome Pathways—Opportunities and Challenges for Endocrine Disrupting Chemical Identification

**FIGURE 1:**
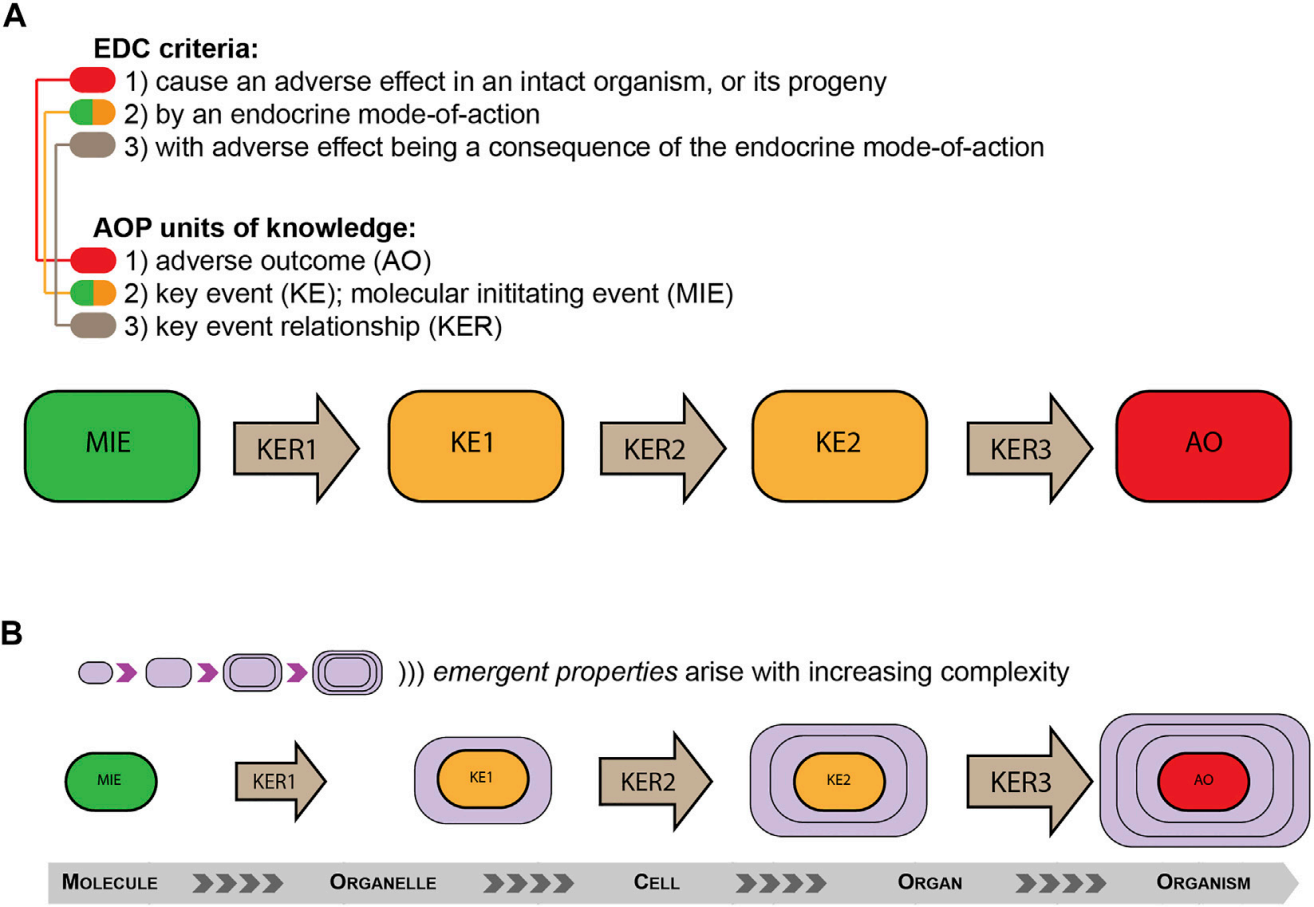
Identifying Endocrine Disrupting Chemicals (EDCs) using the Adverse Outcome Pathway (AOP) concept. **(A)** Current criteria for the identification of, for instance, pesticides and biocides with endocrine disrupting properties (ECHA/EFSA, 2018) aligns with the basic building blocks comprising an AOP; from the molecular initiating event (MIE) or key events (KEs) through to adverse outcomes (AOs), which are linked by key event relationships (KERs) that infer causality. **(B)** Emergent properties refer to entirely new and unexpected properties that arise as complexity of a system increases. Although KERs in principle can incorporate knowledge that allows for predicting also emergent properties, they remain exceedingly difficult to predict at the higher levels of complexity, as they do not belong to any one part of the system.

Much has been written about the AOP concept and it is not my intent to repeat it here. For the interested reader, some reviews can be recommended as a starting point (Ankley et al., 2010; Villeneuve et al., 2014; Solecki et al., 2017; Vinken et al., 2017; Carusi et al., 2018; Svingen et al., 2021). Here, it will suffice to say that AOPs are, in principle, simplified causal pathways linking molecular initiating event (MIE), e.g., the binding of a chemical substance to a nuclear receptor, with an adverse outcome (AO). An AOP aims to only include key events (KEs) that are both measurable and essential for progression down the causal (toxicologically relevant) pathway. Finally, individual KEs are linked by key event relationships (KERs), which essentially represents the knowledge of biological inference in any causal AOP. What becomes immediately apparent from this AOP structure, is that it matches quite well that of the WHO criteria for defining an EDC, and even more so the ECHA/EFSA guidance for assessing pesticides (ECHA/EFSA, 2018), as depicted in Figure 1A. To reiterate, for a chemical substance to fulfil the EDC criteria, it should 1) cause an adverse outcome in an intact organisms (which would correspond to an AO), by 2) an endocrine mode of action (which would correspond to a MIE), by 3) a biologically plausible relationship (which would correspond to a KER).

The other point that becomes apparent with the AOP approach to risk assessment is the increasing complexity as one moves from an MIE towards and AO. As depicted in Figure 1B, the concept of emergent properties can easily be appreciated in AOP pathways. For every incremental step down the causal pathway, the biological complexity increases and surpasses the complexity of the sum of the previous events. Thus, predicting the true nature of a downstream state based on knowledge from upstream events only, can become exceedingly difficult, so much so that when the complexity becomes truly large, wholly accurate predictions becomes impossible. This is further complicated by the fact that future use of AOPs for risk assessment purposes will most likely have to rely on the combination of several complex AOPs, so called AOP networks that may also involve branching AOPs where different causal pathways intercept and interact to cause adverse outcomes (Sewell et al., 2018). Although the second complexity does not necessarily reflect the issue of emergent properties, when combined these issues represent a great challenge with the current effort of replacing animal studies with NAMs for predictive toxicology. This does not mean that we should not try, but we have to be reasonable and rational. What plays in our favor, however, is that we do not have to predict adverse effect in intact organisms (e.g., humans and wildlife) with hundred percent accuracy in order to assess the potential for chemical substances to cause harm, and subsequently regulate based on this knowledge. How good the predictions have to be to reasonably safeguard human health, however, remains a debatable question and something that must be properly integrated in future risk assessment strategies. Because the fact remains, that in many instances the predictive power remains quite poor, even for well-defined endocrine disrupting modalities such as “androgenic” and male reproduction (Svingen et al., 2022).

## Concluding Remarks

Regulatory toxicology is moving towards greater reliance on NAMs to identify, assess and regulate potential harmful chemical substances. This will greatly reduce the number of animals used for regulatory testing purposes and, at the same time, speed up at the process of chemical assessment. This is a positive thing and a widely shared sentiment. However, if we are to also protect human health or the environment from possible harm caused by unintentional, or unwitting, exposure to industrial chemicals with potential to cause injurious health effects, then we should also consider the many limitations with new approaches. This should not be a *one-or-the-other* debate that divides proponents for different approaches into trenches from where they can engage in intellectual warfare. Rather, it would be best served with a *one-and-the-other* debate, where there is a gradual transition phase best suited for whatever area of health effects or regulatory toxicology being adressed.

Thus, although I am convinced we will make great strides towards improving the predictive power of NAMs over the next decade, I will end with another quote from P.W. Anderson’s classic “*More is Different*” paper, as it so elegantly summarizes the potential pitfall of assuming that any simplified model can truly capture the essence of complex systems: “*The constructionist hypothesis does not and cannot live up to its promise since the reduction on which it is based had not included the equally fundamental fact that “entirely new properties*” *arise at each new level of complexity and scale*” (Anderson, 1972). Again, this is not to sound discouraging, but simply—in my opinion—realistic.

## Data Availability

The original contributions presented in the study are included in the article/Supplementary Material, further inquiries can be directed to the corresponding author.
